# Engineering Sub-Nanometer Channels in Two-Dimensional Materials for Membrane Gas Separation

**DOI:** 10.3390/membranes8040100

**Published:** 2018-10-29

**Authors:** Liang Huang, Haiqing Lin

**Affiliations:** Department of Chemical and Biological Engineering, University at Buffalo, The State University of New York, Buffalo, NY 14260, USA; lhuang28@buffalo.edu

**Keywords:** graphene oxide, two-dimensional materials, membranes for gas separation, mixed-matrix materials

## Abstract

Sub-nanochannels constructed by stacking two-dimensional (2D) nanosheets in parallel provide a unique molecular separation pathway with excellent size-sieving ability for membrane gas separation. Herein we review the progress in engineering these 2D channels for efficient gas separation including graphene, graphene oxide (GO), molybdenum disulfide (MoS_2_), and MXene. Mixed matrix materials containing these 2D materials in polymers are also reviewed and compared with conventional polymers for gas separation.

## 1. Introduction

Compared with conventional distillation, absorption, and adsorption processes, membrane-based gas separation is inherently compact, easy to operate, and energy-efficient [1,2]. Membranes are practiced for nitrogen production from air, natural gas processing (CO_2_/CH_4_ separation), and H_2_ purification and recovery (H_2_/CO, H_2_/CH_4_, and H_2_/N_2_ separation), and they are also explored for CO_2_ capture from flue gas (CO_2_/N_2_ separation) and syngas (H_2_/CO_2_ separation) [2,3]. Commercial gas separation membranes are mainly fabricated from polymers because of their excellent processibility, and easy production scale-up [3]. However, polymers are limited by a trade-off between permeability and selectivity, i.e., polymers with higher permeability often exhibit lower selectivity, and vice versa [2,3,4,5]. The upper bound of membrane gas separation has been summarized by Robeson [2,4] and rationalized using a transition-state theory and free volume model [6,7]. One key challenge to advance membrane technology is to develop materials with both high permeability and selectivity.

The emerging two-dimensional (2D) layered materials have provided an unprecedented opportunity for the development of high-performance membrane materials [8,9,10]. These 2D nanosheets can be stacked in parallel into ultrathin membranes, forming the sub-nanometer channels between the sheets with the molecular size-sieving ability [11,12]. Among all the 2D materials fabricated such as graphene, MoS_2_, carbides, and nitrides (MXenes), graphene and graphene oxide (GO) attracted a lot of attention and have been intensively studied for membrane gas separation because of its easy chemical functionalization and tunable gap size. Without exhaustively reviewing the status of all 2D materials-based membranes (such as clay materials), this short report summarizes some of the key progresses in manipulating the sub-nanometer channels in 2D materials for membrane gas separation with an emphasis on GO-based materials. First, the gas permeability of pristine graphene is discussed. Second, the methods of tuning the size of the nanochannels to enhance the gas separation properties in the GO-based membranes are reviewed and compared. Third, membranes based on other 2D materials for gas separation are discussed. Finally, mixed matrix membranes (MMMs) comprising 2D materials dispersed in polymers (to improve the processibility) or intercalated with CO_2_–philic materials (to enhance CO_2_ permeability) for gas separation are reviewed.

## 2. Graphene-Based Membranes

### 2.1. Gas Permeability of Graphene and GO Nanosheets

**Figure 1:**
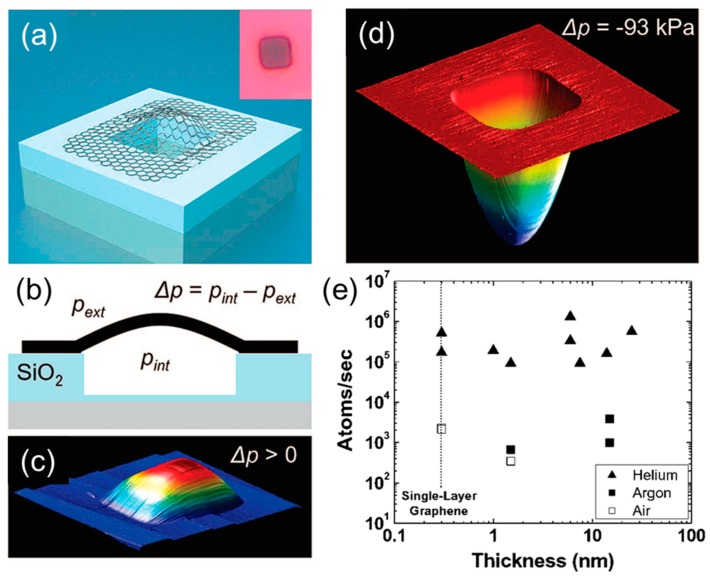
(**a**) Schematic of a microchamber sealed by a graphene drumhead. Inset is the optical image of graphene sealed SiO_2_ microchamber with dimensions of 4.75 µm × 4.75 µm × 380 nm. (**b**) Cross-sectional schematic of the graphene sealed microchamber. AFM image of the graphene sealed microchamber with (**c**) *p_int_* > *p_ext_* and (**d**) *p_int_* < *p_ext_*. (**e**) Effect of the thickness of the graphene drumhead on the permeation rates of various gases. Adapted with permission from [14]. American Chemical Society (2008).

Graphene is composed of a single layer of carbon atoms arranged in an sp^2^-bonded aromatic structure [13], which can be regarded as a membrane with one-atom thickness (0.34 nm). Figure 1 shows one of the first studies of gas transport properties in graphene membranes, which was impermeable to various gases [14]. As shown in Figure 1a, a graphene membrane was suspended and clamped over a predefined well in silicon oxide (SiO_2_) using the van der Waals force between graphene and SiO_2_, forming a graphene-sealed microchamber. Graphene membranes are single-layer or multi-layer (2–75) graphene sheets, which were produced by mechanical exfoliation of Kish graphite using Scotch tape [14]. After initial fabrication, the pressure inside the microchamber, *p_int_*, was the same as the atmospheric pressure. As the pressure outside the chamber (*p_ext_*) changed, *p_int_* gradually approached *p_ext_* (Figure 1b). During this period, the membrane was stretched like a balloon (*p_int_* > *p_ext_*, Figure 1c) or deflected inward (*p_int_* < *p_ext_*, Figure 1d). By monitoring the change of *p_int_* with time, the flux of helium, argon, and air were calculated, and they were independent of the graphene thickness (or number of graphene layers), as shown in Figure 1e. The gas flux was also consistent with that through the SiO_2_ well. The authors concluded that the gas flux cannot be ascribed to the permeation through the graphene layers, and even the smallest gas He could not penetrate through the one-atom-thick graphene membrane [14].

Nanopores can be generated on the graphene sheets to allow gas permeation and separation by ultraviolet-induced oxidative etching [15] or focused ion bean (FIB) drilling [16]. Both theoretical and experimental results show that the resulting nanoporous graphene membrane can be highly selective and permeable to gases if the pore size and pore density are well controlled [15,16,17]. However, the fabrication of large-area single-crystal monolayer graphene is too expensive, and the production scale-up of the defect-free graphene membrane is extremely challenging [10]. Moreover, current techniques cannot drill sub-nanometer pores with a uniform size and high density on the graphene sheets at a large scale.

**Figure 2:**
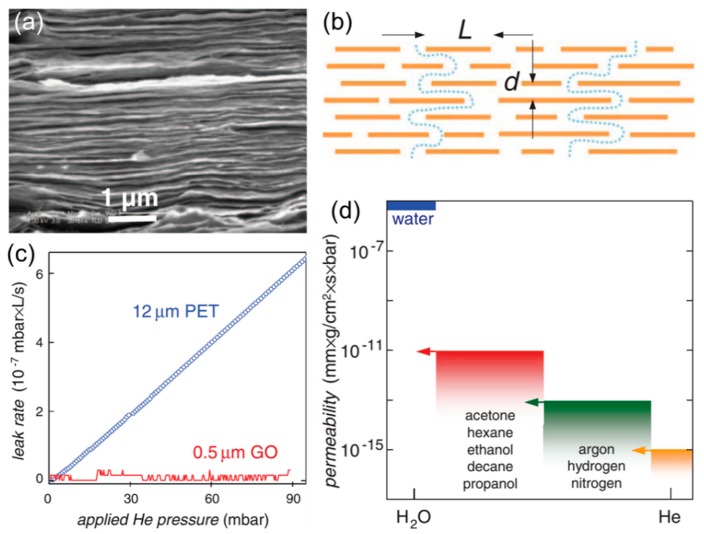
(**a**) Cross-sectional SEM image of a GO film. (**b**) A schematic view of molecular permeation through the GO film. (**c**) He-leak rates in a 0.5-μm GO film and a 12-μm PET film. (**d**) Permeability of water and various small molecules through a GO film. The arrows indicate the upper limit of the permeabilities determined by their experiments [11]. Reprinted with permission from AAAS.

On the other hand, oxidized graphene (or GO) with a high density of oxygen-containing functional groups and few vacancy defects in the carbon lattice can be cheaply produced in a large scale by oxidation and exfoliation of graphite [18,19,20]. The vacancy defects or holes (usually <5 nm^2^) on a GO sheet only occupy ~2% area, and carbonyl groups often form at the edges of the holes [21]. GO sheets are dispersible in water due to their hydrophilicity and the electrostatic repulsion of their ionized functional groups, such as carboxyl groups [22]. GO sheets usually have a thickness of ~1 nm and lateral dimension ranging from several hundred nanometers to several micrometers. Ultrathin GO laminar membranes can be prepared by vacuum filtration [12,23], spin coating [1], and drop-casting [11], as shown in Figure 2a. The sub-nanometer 2D channels between the adjacent GO layers exhibit strong size-sieving ability, resulting in superior gas separation properties.

In 2012, Geim and coworkers observed the unimpeded permeation of water vapor through submicrometer-thick GO membranes for the first time [11]. Water molecules permeate through the interlayer nanochannels between adjacent GO sheets (Figure 2b). Interestingly, dry GO membranes were completely impermeable to other vapors and gases (Figure 2c,d) [11]. For example, helium permeability is lower than ~10^−15^ mm·g/cm^2^·s·bar or 7.5 × 10^−5^ Barrer (1 Barrer = 10^−10^ cm^3^ (STP) cm/cm^2^·s·cmHg) (Figure 2d). However, in the presence of water vapor, the GO membranes exhibit much higher He permeability due to the permeation through the water between the GO sheets.

### 2.2. Engineering the Nanochannels between GO Nanosheets

In contrast to the observation by Geim and coworkers [11], the dry GO membranes were reported to be permeable to gases at transmembrane pressures higher than a critical pressure to overcome the energy barriers for pore entry [1]. For example, GO membranes (4~6 μm) were prepared by vacuum filtration and exhibited gas permeability decreasing in the same order as the increase of the molecular size: He > H_2_ > CO_2_ > O_2_ > N_2_ > CH_4_ [1]. Larger GO sheets lead to lower gas permeability and higher critical transmembrane pressures due to the more extended diffusion paths.

**Figure 3:**
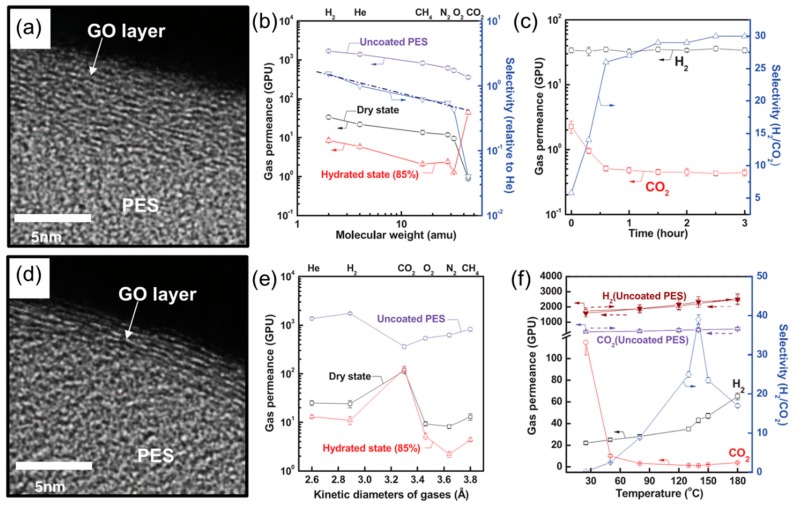
The structure and gas transport behavior of GO membranes prepared by dip and spin-coating (**a**–**c**). (**a**) Cross-sectional TEM image. (**b**) Gas permeance as a function of molecular weight under dry and humidified conditions. The dashed line represents the ideal Knudsen selectivity. (**c**) H_2_/CO_2_ separation properties as a function of time. The structure and gas transport behavior of GO membranes prepared by drop and spin-coating (**d**–**f**). (**d**) Cross-sectional TEM image. (**e**) Gas permeances as a function of gas kinetic diameters under dry and humidified conditions. (**f**) H_2_/CO_2_ separation properties as a function of temperature. 1 GPU = 10^−6^ cm^3^ (STP)/cm^2^·s·cmHg. The dashed arrows indicate the testing temperatures increasing from 25 to 180 °C and then back to 25 °C. Reprinted with permission from AAAS.

Ultrathin GO membranes (3 to 10 nm) were prepared by depositing GO on commercial microporous polyethersulfone (PES) membranes in two methods, dip and spin-coating and drop and spin-coating. Figure 3a shows the TEM image of the membranes derived from the first method, where nanopores are formed at the edges of less interlocked GO sheets [1]. Except for CO_2_, most gases showed typical Knudsen diffusion behavior (Figure 3b), i.e., gas permeance decreases proportionally with decreasing Mw^−0.5^. For example, the membrane showed a H_2_/CO_2_ selectivity of 30 (Figure 3c), which is much higher than the theoretical Knudsen selectivity (4.7). The unexpectedly low CO_2_ permeance may be caused by the strong interaction between CO_2_ and the carboxyl groups at the edge of the GO sheets and the resulting impedance for CO_2_ diffusion. Interestingly, gas permeances decreased with increasing the humidity in the feed gas except for CO_2_. The water between GO sheets blocked the transport of most gases while facilitating the interaction between CO_2_ and carboxyl groups and thus CO_2_ diffusion, leading to a ~50 times increase in the CO_2_ permeance (Figure 3b).

Figure 3d shows the GO membranes prepared by the drop and spin-coating method, which are denser and more ordered than those prepared using the dip and spin-coating method. The gas transport through the resulted membranes follows the solution-diffusion mechanism, instead of the Knudsen diffusion (Figure 3b). The presence of water vapor in the feed had minimal effect on CO_2_ permeance and significantly decreased the permeance for other gases (Figure 3e). In addition, thermal treatment of the GO membranes generated nanopores on the basal plane of the GO sheets, increasing the H_2_ permeance. The thermally annealed GO membranes exhibited a H_2_/CO_2_ selectivity as high as 40 at 140 °C (Figure 3f).

**Figure 4:**
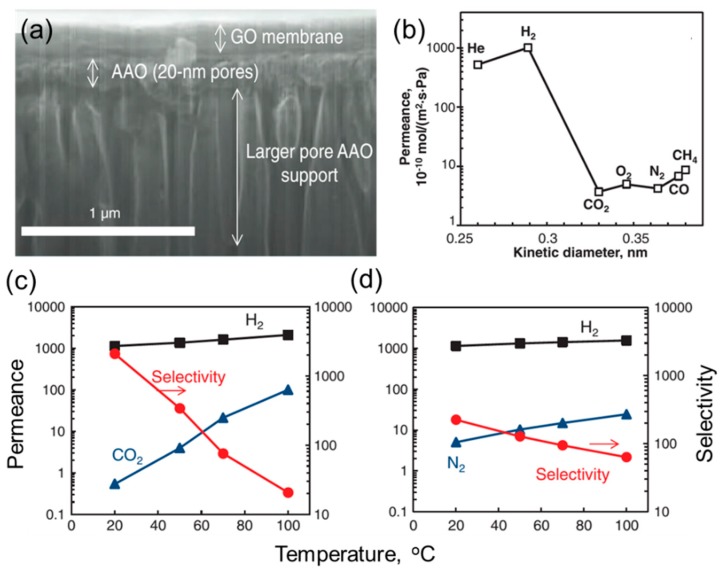
(**a**) Cross-sectional SEM image of a GO membrane (~180 nm) supported on porous AAO. (**b**) Gas permeances of a ~18-nm-thick GO membrane at 20 °C. Effect of temperature on mixed-gas (50:50) (**c**) H_2_/CO_2_ and (**d**) H_2_/N_2_ separation properties in a 1.8-nm-thick GO membrane. From [12]. Reprinted with permission from AAAS.

Ultrathin GO membranes were also deposited on anodic aluminum oxide (AAO) films by vacuum filtration (Figure 4a) and showed extraordinary gas separation performance [12]. As shown in Figure 4b, an 18-nm-thick GO membrane exhibited H_2_/CO_2_ selectivity of 300. Interestingly, gas permeance decreased exponentially with the thickness of the GO membrane, instead of the inverse thickness for conventional membranes. Such unexpected gas transport properties were ascribed to the dominant transport pathway being the selective structural defects within the GO sheets, instead of the interlayer spacing between GO sheets [12]. The thinnest GO membrane prepared was only 1.8 nm, which corresponds to two layers of GO sheets. In the mixed gas tests at 20 °C, this 1.8-nm-thick GO membrane exhibited an unprecedented H_2_/CO_2_ selectivity of ~2500 and a H_2_/N_2_ selectivity of ~200, along with a high H_2_ permeance of ~300 GPU (Figure 4c,d). As a comparison, the GO membranes (3−7 nm) reported by Kim et al. showed a H_2_ permeance of 35 GPU and H_2_/CO_2_ selectivity of 30 (Figure 3c). The significant discrepancy between these two reports suggests that the molecular separation performance of the GO membranes depends sensitively on the stacking mode and structure of GO sheets, such as lateral size, defects within GO sheets, and oxidation degree. Nevertheless, these separation properties are very attractive for H_2_/CO_2_ separation for H_2_ purification and CO_2_ capture. However, it would be beyond the scope of this study to investigate the optimal membrane process designs and targeted membrane separation properties.

**Figure 5:**
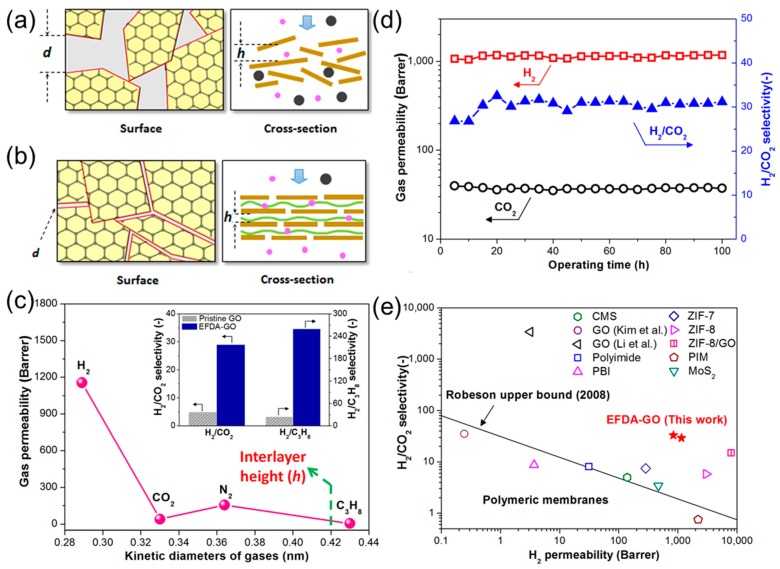
Schematic of GO-based 2D channels with (**a**) disordered structure and (**b**) highly ordered laminar structures. (**c**) Gas permeability of the EFDA−GO membranes. The inset compares the EFDA−GO with pristine GO membrane for H_2_/CO_2_ and H_2_/C_3_H_8_ selectivity. (**d**) Long-term H_2_/CO_2_ separation performance of the EFDA−GO membranes. (**e**) Comparison of the EFDA−GO membranes with the state-of-the-art materials for H_2_/CO_2_ separation. Adapted with permission from [24]. American Chemical Society (2016).

The GO membranes described above were assembled through intrinsic forces including van der Waal’s force, hydrogen bonding, and electrostatic interaction. The assembly of GO nanosheets can also be prepared using external forces such as compressive and shear force, and molecular interaction between GO and molecular binders [24]. For example, GO membranes were prepared by alternative vacuum-spin coating of a GO solution (to control both compressive and shear force) and polyethyleneimine (PEI) solution (as a binder for the GO nanosheets). The as-produced external force driven assembled GO (EFDA-GO) membranes exhibited highly ordered layer structure with 2D channels of ~0.4 nm (Figure 5b), resulting in strong size-sieving ability. The H_2_/CO_2_ and H_2_/C_3_H_8_ selectivity were 6 and 11 times higher than that of the pristine GO membranes, respectively (inset of Figure 5c). With a H_2_/CO_2_ selectivity of ~30, the EFDA−GO membranes showed a H_2_ permeability as high as 1000 Barrers (Figure 5d,e), which is almost 300 times higher than that reported by Li et al. [12] and about 5000 times higher than that reported by Kim et al. [1]. As shown in Figure 5e, the EFDA−GO membranes exhibited much more superior H_2_/CO_2_ separation properties than the leading polymers for this application such as polybenzimidazole (PBI) and polyimide (PI), and well above the Robeson’s 2008 upper bound [24].

**Figure 6:**
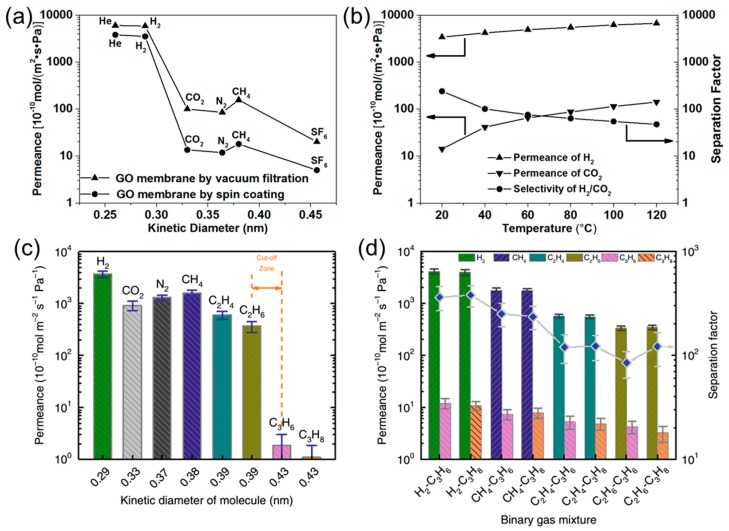
(**a**) Comparison of gas permeances of GO membranes fabricated by spin coating and vacuum filtration. (**b**) Effect of temperature on H_2_/CO_2_ separation performance of the spin-coated GO membranes. Adapted with permission from [25]. Copyright (2016) American Chemical Society. (**c**) Pure-gas and (**d**) mixed-gas permeances through the ED-GO membranes at room temperature. Adapted with permission from [26].

To obtain GO sheets with uniform dimension, GO sheets with a uniform size were prepared by mild freeze-thaw exfoliation and pH adjustment, instead of commonly used sonicating exfoliation [25], because sonication may break GO sheets into smaller pieces with a wide size distribution. The GO sheets were deposited on an AAO substrate by vacuum filtration, or by spin-coating at 90 °C with rapid water evaporation (which matches the GO deposition rate to obtain high-quality membranes) [25]. Figure 6a demonstrates that gas permeability decreased with increasing molecular size of the gases. The spin-coated membranes showed lower gas permeance but much higher H_2_/CO_2_ selectivity (259) than that prepared by vacuum filtration (58), because the wrinkles and defects in the membranes prepared by vacuum filtration served as additional pathways [25]. As shown in Figure 6b, increasing the temperature increased the gas permeance and decreased the H_2_/CO_2_ selectivity, which is consistent with other literature studies [1,12].

The nanochannels in GO membranes can be tuned by controlled reduction or cross-linking to achieve targeted separation. When an ultrathin GO was deposited on porous stainless steel hollow fibers by electrophoresis deposition (ED) [26], the oxygen-containing functional groups on GO sheets were removed, decreasing the gap of the 2D nanochannels (~0.36 nm). The ED-GO membranes exhibited a sharp cutoff between C2 and C3 hydrocarbons (Figure 6c) and C2/C3 selectivity as high as 100 in the mixed-gas tests (Figure 6d). Interestingly, these membraned did not show high H_2_/CO_2_ selectivity. GO membranes with narrowed 2D nanochannels (~0.34 nm) were also prepared via crosslinking GO sheets with thiourea [27]. The resulting membranes (with a thickness of ~350 nm) showed an unprecedented H_2_ permeance of ~2000 GPU and H_2_/CO_2_ selectivity of ~200 [27].

## 3. Gas Separation Membranes Based on Other 2D Materials

Besides graphene and GO, other 2D materials like MoS_2_ [28] and MXenes [29,30] have also been fabricated into membranes for gas separation. Single- or few-layered MoS_2_ nanosheets can be prepared from bulk MoS_2_ flakes by sonication or chemical exfoliation. The as-prepared MoS_2_ nanosheets are dispersible in water and can be easily assembled into layered MoS_2_ membranes by vacuum filtration [28]. A 60-nm MoS_2_ membrane exhibited a H_2_ permeance of 1768 GPU and a H_2_/CO_2_ selectivity of 4.4 (close to Knudsen diffusion selectivity of 4.7), presumably because the interlayer spacing in MoS_2_ membranes is too large for the separation of gas molecules.

MXenes are a family of 2D transition metal carbides and nitrides, which can be produced by selectively etching the A layer (mostly group IIIA or IVA elements, such as Al) from the layered M_n+1_AX_n_ phase ceramics [31]. M stands for an early transition metal, X refers to carbon and/or nitrogen, and n equals 1, 2, or 3. For example, Ti_3_AlC_2_ is a typical M_n+1_AX_n_ phase ceramic. So MXenes have a formula of M_n+1_X_n_T_x_, where T_x_ are functional groups (such as –O–, –OH and –F) grafted on the surface of MXene nanosheets during the etching and exfoliation processes. These functional groups on MXene nanosheets make them hydrophilic and highly dispersible in water, and therefore, they are excellent building blocks to construct ultrathin membranes. The functional groups also offer active sites for chemical functionalization to tune the size and chemical properties of the nanochannels.

**Figure 7:**
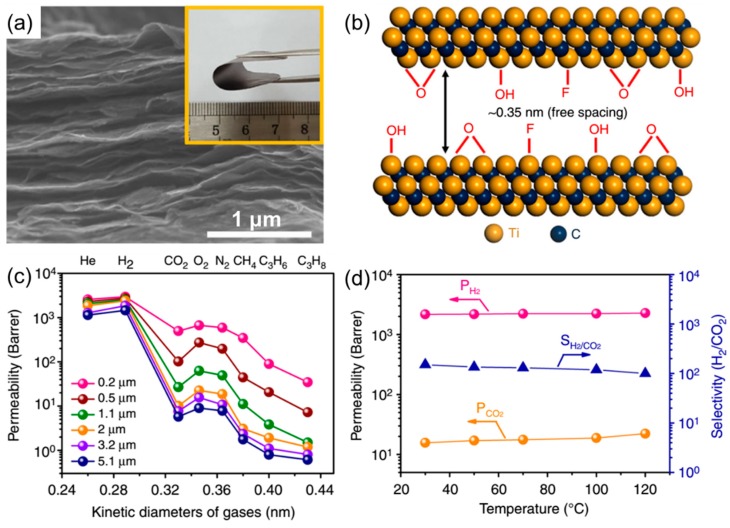
(**a**) Cross-sectional SEM image of a MXene membrane. Inset is a bent membrane. (**b**) Illustration of a nanochannel between two nanosheets. (**c**) Effect of the film thickness on the gas permeability of MXene membranes. (**d**) Mixed-gas H_2_/CO_2_ separation performance of a 2-μm-thick MXene membrane at 30–120 °C. Adapted with permission from [30].

Figure 7a shows an MXene membrane prepared by vacuum filtration of Ti_3_C_2_T_X_ nanosheets on an AAO support. Ti_3_C_2_T_X_ nanosheets were fabricated by selectively etching Al from the Ti_3_AlC_2_ phase using hydrochloric acid and lithium fluoride [30]. The nanochannels between the nanosheets are estimated to be 0.35 nm (Figure 7b), which is desirable for gas separation. Figure 7c demonstrates an unexpected behavior, i.e., gas permeability decreased with increasing the film thickness, presumably because the thin MXene layers (<2 μm) contain defects, and the defects decreased with increasing thicknesses. Figure 7d shows that a 2-μm-thick MXene membrane showed both high H_2_ permeability (~2000 Barrers) and high H_2_/CO_2_ selectivity (>100) at 30–120 °C, which is comparable with the best GO membranes reported so far [27].

## 4. MMMs Containing 2D Nanochannels for Gas Separation

One of the challenges in developing 2D materials-based membranes is the production of large-scale membranes (in 1000 m^2^ or more) with good reproducibility and low cost. One solution is to hybridize 2D materials with organic materials (such as polymers) with good processibility. Two approaches have been widely explored, intercalating organic materials between 2D layers, and dispersing 2D layers in polymers. A comprehensive review of the MMMs can be referred to a recent paper [32]. This report highlights some of the key strategies utilized to design and prepare MMMs, achieving interesting membrane gas separation properties.

### 4.1. 2D Layers Intercalated with CO_2_-Philic Materials

**Figure 8:**
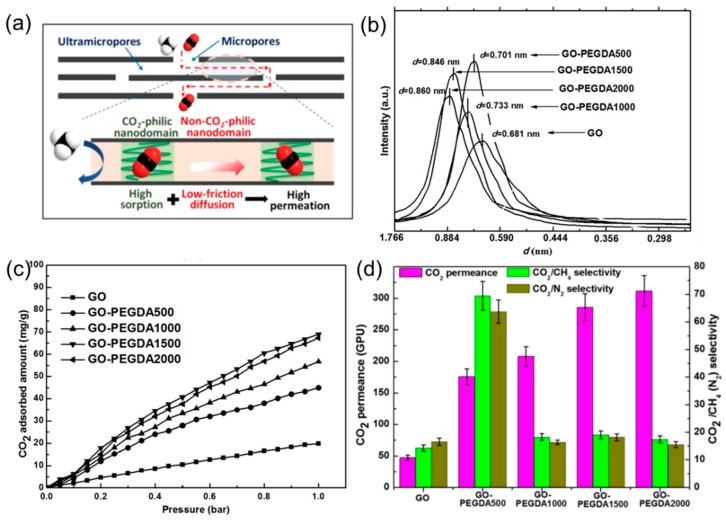
(**a**) Schematic of gas transport in GO membrane channels composed of both CO_2_-philic and non-CO_2_-philic nanodomains. (**b**) XRD patterns of GO and GO-PEGDA samples with the corresponding d-spacings. (**c**) CO_2_ sorption isotherms of the membranes. (**d**) Mixed-gas separation performances of the membranes tested in the dry state at 2 bar and 30 °C (50/50 vol% CO_2_/N_2_ or CO_2_/CH_4_). Adapted with permission from [34]. John Wiley and Sons, Inc.

GO membranes usually show low CO_2_ permeability, which limits their applications for CO_2_ capture. On the other hand, GO layers can be intercalated with CO_2_–philic materials to facilitate CO_2_ transport, such as polyethylenimine [24], borate [33], poly(ethylene glycol) diamines (PEGDA) [34], ionic liquids [35], and piperazine [36]. Figure 8a presents the intercalation of GO sheets using PEGDA, whose amine groups react with the epoxy groups on the surface of GO sheets, forming CO_2_-philic nanodomains [34]. The intercalation increased the size of the GO nanochannels (Figure 8b), and the CO_2_ sorption (Figure 8c). For instance, the GO-PEGDA2000 absorbed about 3 times more CO_2_ than the pristine GO, and it exhibited a CO_2_ permeance of 300 GPU, which is ~5 times higher than that of the pristine GO membrane (47 GPU). The GO-PEGDA500 shows a CO_2_/CH_4_ selectivity of ~70, which is much higher than the GO membrane (14). As shown in Figure 8d, the CO_2_ permeance increased with increasing PEGDA molecular weight presumably because of the enlarged channel size. On the other hand, the highest CO_2_/CH_4_ and CO_2_/N_2_ selectivity is achieved by GO-PEGDA500 membranes because of their proper channel size (~0.35 nm), which is right between the kinetic diameter of CO_2_ (0.33 nm) and CH_4_ (0.38 nm) or N_2_ (0.364 nm).

Similar to the GO membranes, MXene can also be chemically modified for the selective transport of CO_2_. For example, the CO_2_-philic borate and PEI were intercalated between the MXene nanosheets, which transformed the diffusion-controlled H_2_-selective channels to solution-controlled CO_2_-selective ones [29].

### 4.2. MMMs Containing GO in Polymers for Gas Separation

In general, GO layers exhibit lower gas permeability and stronger size-sieving ability than most polymers. Therefore, the incorporation of the GO in highly permeable polymers often decreases gas permeability. For example, incorporating 8 wt% of GO in polydimethylsiloxane (PDMS) resulted in a 99.9% reduction in permeability for various gases, such as H_2_, CO_2_, N_2_ and CH_4_ [37]. The diffusion of larger molecules (N_2_ and CH_4_) is reduced more than that of smaller molecules (H_2_ and CO_2_). As a result, CO_2_/N_2_ and CO_2_/CH_4_ selectivity increased by a factor of 2‒3. Incorporating 4 wt% GO in cross-linked poly(ethylene oxide) (XPEG) also decreased gas permeability by 80–90%, which was interpreted using Nielson’s model [38]. Nevertheless, these GO-based MMMs seem to be more suitable for polymers with low gas permeability, where the gas diffusion in the 2D channels can be dominant.

**Figure 9:**
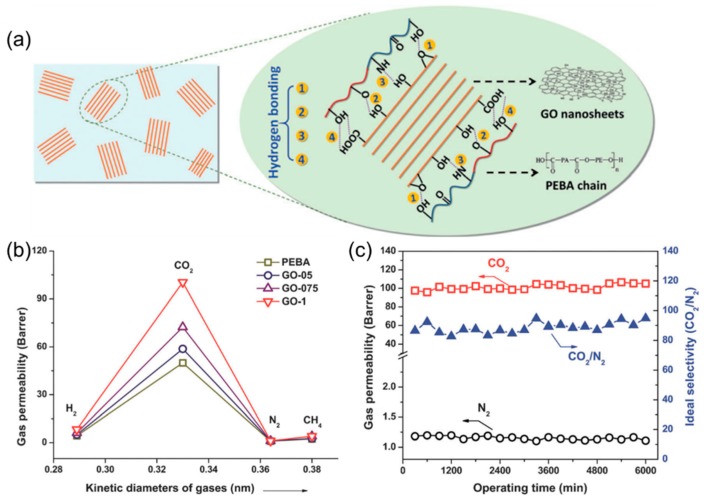
(**a**) Schematic of embedding GO nanosheets in PEBA. (**b**) The gas permeability of the MMMs with different GO loadings (0.05, 0.075, and 0.1 wt%). (**c**) Long-term stability test of an MMM containing 0.1 wt% GO for CO_2_/N_2_ separation. Adapted with permission from [39]. John Wiley and Sons, Inc.

Figure 9a shows the MMMs containing GO in poly(ether-block-amide) (PEBA), which exhibited both higher CO_2_ permeability and CO_2_/N_2_ selectivity than PEBA [39]. The GO nanosheets can be dispersed in the PEBA due to the hydrogen-bonding interactions and assemble into few-layered GO laminates, which had thicknesses ranging from 6 to 15 nm and an interlayer distance of 0.7 nm or a channel of 0.35 nm (after excluding the thickness of graphene). Therefore, these few-layered GO laminates can provide selective transport pathways for CO_2_ over N_2_ and CH_4_ (Figure 9b). Furthermore, the GO-1 membrane was reduced by heating at 150 °C to narrow the interlayer spacing, which decreased the CO_2_ permeability from 100 to 66 Barrers and had negligible effect on the N_2_ permeability. This result suggests that N_2_ molecules cannot diffuse through the interlayer spacing of GO laminates, and the enhancement in the CO_2_ permeability is due to the presence of the GO, instead of the change of the polymer phase caused by the GO dispersion. Interestingly, the H_2_ permeability did not increase with increasing the GO content in the MMMs, though H_2_ molecules should also be able to diffuse in the GO nanochannels. Figure 9c shows the long-term stability and superior CO_2_/N_2_ separation properties of the MMM containing 0.1 wt% GO.

**Figure 10:**
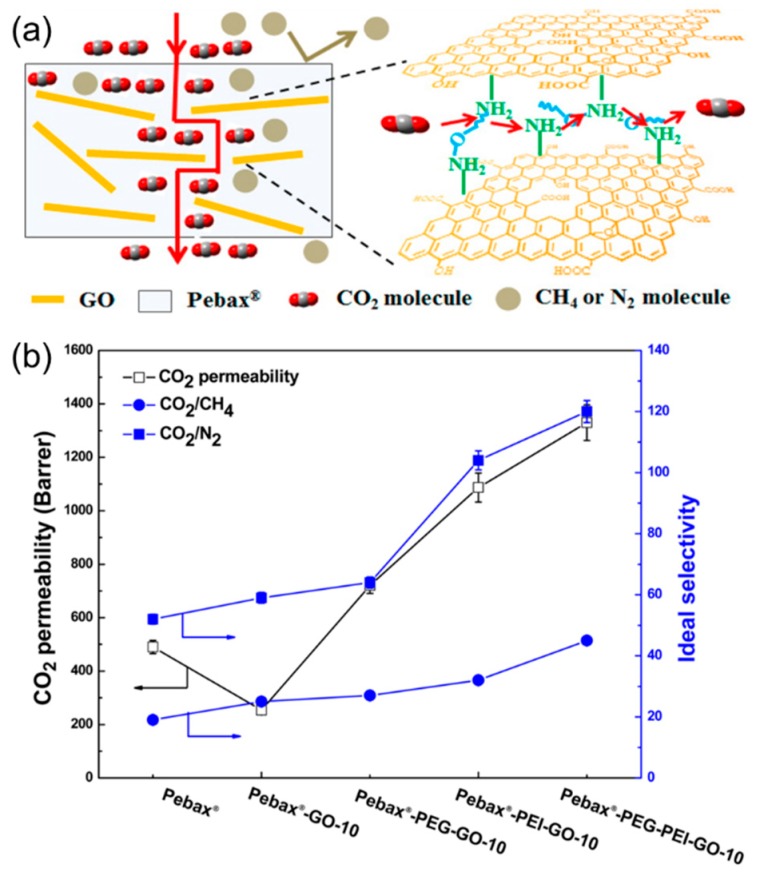
(**a**) Schematic of CO_2_-facilitated transport in the MMMs containing the modified GO in PEBA. (**b**) CO_2_/gas separation properties of PEBA and MMMs containing 10 wt% fillers in the humidified condition. Adapted with permission from [40]. American Chemical Society (2015).

To further enhance the CO_2_ transport, the GO nanosheets can be grafted with CO_2_-philic PEG and PEI and then embedded into PEBA (Figure 10a) [40]. As shown in Figure 10b, incorporating 10 wt% GO in PEBA improved CO_2_/gas selectivity and reduced CO_2_ permeability. However, embedding PEG-modified GO (PEG-GO), PEI-modified GO (PEI-GO) or PEG and PEI modified GO (PEG-PEI-GO) improved both CO_2_/gas selectivity and CO_2_ permeability. For example, an MMM containing 10 wt% PEG-PEI-GO exhibited an ideal CO_2_/N_2_ selectivity of 120, CO_2_/CH_4_ selectivity of 45, and CO_2_ permeability of 1330 Barrers, which is much higher than PEBA.

Although embedding GO nanosheets in polymers has been widely demonstrated to improve gas separation performance [41,42,43,44,45], industrial thin film composite (TFC) membranes with a thin selective layer (~100 nm) have not been reported yet, presumably because of the relatively large lateral dimension of GO nanosheets (from a few hundred nanometers to several micrometers), and the defects generated during the fabrication of the membranes. Aligning GO nanosheets in the polymer to prepare defect-free TFC membranes to achieve high permeance is still challenging.

## 5. Conclusions and Perspective

We review ultrathin membranes based on the stacked 2D nanosheets (including graphene, GO, MoS_2_, and MXene) with promising gas separation performance. GO is the most widely studied 2D materials for membrane gas separation, and its separation performance depends sensitively on the stacking mode of GO sheets, which is influenced by the dimension and structure of GO nanosheets and the preparation method. The nanochannels in the GO membranes can be tuned by the controlled reduction or cross-linking. The 2D materials can also be fabricated into the MMMs to improve the mechanical properties, processibility, and even gas separation properties. For example, the GO layers can be intercalated with CO_2_-philic materials to enhance the CO_2_/gas separation performance, and the incorporation of the GO in PEBA yields superior CO_2_/N_2_ separation properties. However, the challenge faced with these 2D materials-based membranes is their production on a large scale with superior separation properties and good reproducibility. In addition, the effect of the amount and type of the oxygen-containing functional groups on GO nanosheets on gas separation performance is yet to be elucidated.
